# The Highest Cited Papers in Brucellosis: Identification Using Two Databases and Review of the Papers' Major Findings

**DOI:** 10.1155/2018/9291326

**Published:** 2018-04-11

**Authors:** Faris Ghalib Bakri, Hamzah M. AlQadiri, Marwan Hmoud Adwan

**Affiliations:** ^1^Infectious Disease and Vaccine Center, The University of Jordan, Amman, Jordan; ^2^Department of Medicine, Division of Infectious Diseases, School of Medicine, The University of Jordan, Amman, Jordan; ^3^Department of Nutrition and Food Technology, School of Agriculture, The University of Jordan, Amman, Jordan; ^4^Department of Medicine, Division of Rheumatology, School of Medicine, The University of Jordan, Amman, Jordan

## Abstract

Citation classics represent the highest impact work in a given field. We aim to identify and analyze the most frequently cited papers on brucellosis. We used the databases Scopus and Web of Science to determine the most frequently cited papers. The most cited fifty papers in each database were identified. We then ranked the papers according to the highest citation count recorded from any of the two databases. The most frequently cited paper received 964 citations and was by DelVecchio VG et al. reporting the complete genomic sequencing of* Brucella melitensis*. The papers were published in 30 journals led by the “Infection and Immunity” journal and the “Veterinary Microbiology” journal (each had 7 papers). Citation classics in brucellosis were all in English except one in French and were mostly of basic science type. In addition, we noticed that 12 articles that were identified among the highest fifty articles in one database were missed by the other database and vice versa. Therefore, we suggest that searching in more than one database would detect additional citation classics.

## 1. Introduction

Brucellosis is a zoonotic granulomatous disease that can affect any organ. It is caused by* Brucella* species which are small, Gram-negative, and coccobacilli bacteria. Clinical presentation varies from an acute, nonspecific febrile illness to chronic, debilitating forms with features of osteoarticular and neuropsychiatric abnormalities [1].

Brucellosis was first described in 1887 by David Bruce, a British surgeon, who isolated Gram-negative coccobacilli from the spleens of five British soldiers who died of fever in Malta. In 1905, Zammit, a Maltese bacteriologist, showed that infected goats transmitted brucellosis and that banning the use of their milk would be effective in eliminating the disease. The observation that apparently healthy goats could be carriers of the disease has been termed one of the greatest advances ever made in the study of epidemiology [2].

The disease has wide geographic distribution and it is one of the most economically important zoonosis. In a review of 76 diseases of animals, brucellosis lies within the top 10 in terms of impact on poor people [3]. In low-income countries, brucellosis is endemic and neglected. It also causes large disease in animals and people and lacks effective control [1, 4–6]. Accurate epidemiological data are not available for many endemic areas, but it has been estimated that more than 500,000 new human cases occur annually [7].

In 1987, Garfield listed the “top 100” best cited articles ever published in JAMA and named them “citation classics” [8], and these classics represent the highest impact work in a given field [9]. Citation analysis in the field of infectious diseases and microbiology was reported for tuberculosis [10], nontuberculous mycobacteria [11], anthrax [12], Severe Acute Respiratory Syndrome (SARS) [13], JC virus [14], herpes simplex virus [15], Ebola virus [16], schistosomiasis [17], sepsis [18], and neglected infectious diseases [19]. Here, for the first time to our knowledge, we identify and analyze the citation classics for brucellosis.

## 2. Materials and Methods

Two electronic databases, Scopus and Web of Science (WOS), were searched for the 50 most cited articles using the keyword “brucell^*∗*^.” For the search in Scopus, we selected the “title, abstract, keyword” choice. For the search in WOS, we selected the “topic” and “all database” choices. The search in both databases was performed on January 30, 2017, for papers published in all times. Textbooks were excluded. The most fifty cited papers were identified in both databases. The articles' abstracts were read by the two study investigators (FGB and MHA) to determine whether the articles were specific to brucellosis [20].

We recorded the citation count from the two databases for each selected article. For articles that were among the top fifty articles in one database but not in the other, the citation count in the other database for that article was looked up and recorded. We then ranked the articles according to the highest citation count obtained from any of the two databases.

We analyzed the papers according to number of citations, publication year, authors, journal impact factor, country of origin, and article type (basic science, observational study, interventional clinical trial, and review) [21]. Basic science articles included genetic studies [22], in vitro studies, animal studies, or in vivo studies that focused on physiology [23]. Observational studies included case-control studies, case series, and cohort studies. To classify the article type, two study investigators (FGB and MHA) reviewed all articles independently and in cases of disagreement, they discussed the article until consensus was achieved [21]. The most recent impact factor, year 2015, from Journal Citation report was used for analysis. In cases where the journal has continued as a new title, the impact factor of the new title was used in the analysis [21].

## 3. Results

The list of the most cited articles found in the Scopus and WOS searches is shown in Table 1. The list included 62 articles. Of the total articles, 38 articles appeared in both databases within the highest 50 cited articles. However, among the highest 50 cited articles that were identified by the Scopus search, 12 (24%) articles were not among the top 50 articles within the WOS search. Similarly, among the highest 50 articles that were identified by the WOS search, 12 (24%) articles were not among the highest 50 articles within the Scopus search. All articles eventually appeared in both databases except for one article (position 21) which appeared only in WOS. The mean number of the highest citation count per article from any of the two databases was 284.6 citations (SD = 192.7) and the median number was 197.5 (interquartile range = 169 to 314.5).

Articles that were identified within the highest 50 articles in WOS but not in the highest 50 article in Scopus were at positions 7, 8, 11, 21, 22, 29, 41, 49, 51, 55, 56, and 58. The publication year range was 1950–2011 (mean 1991, SD = 18.3). They were all of basic science type except one article with experimental design. While articles that were in the top 50 article search in Scopus but not in the top 50 article search in WOS were at positions 33, 35, 36, 39, 42, 44, 54, 57, 59, 60, 61, and 62. The publication year range was 1988–2010 (mean 1998, SD = 5.7). They were of observational type in 5 articles, review in 4, and basic in 3.

**Figure 1:**
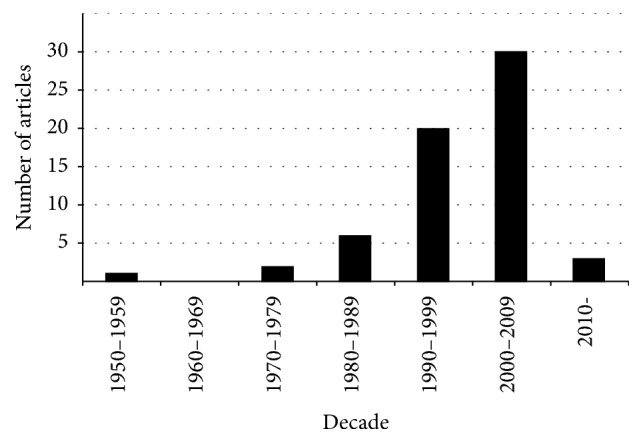
Number of “citation classics” articles according to decade.

The oldest highly cited article was published in 1950 (Harris, JAMA) and the most recent in 2011 (Tae et al., Journal of Bacteriology). The most frequently cited paper received 964 citations (Table 1). The decade from 2000 to 2009 produced the most papers with 30 articles (Figure 1). The most papers published within a given year were 9 in year 2002. Among the citation classics, there were 36 (58%) basic science articles, 16 (26%) review articles, 9 (15%) observational studies, and 1 (1.6%) experimental study.

The papers were all in English except one in French (position 21 by Renoux et al. in 1971). They were published in 30 journals. The median impact factor for journals was 4.32 (range: 1.064 (Comptes Rendus Biologies)–59.558 (New England Journal of Medicine)) (Table 2). Five journals have continued as new titles: Reviews of Infectious Diseases as Clinical Infectious Diseases, International Journal of Systematic Bacteriology as International Journal of Systematic and Evolutionary Microbiology, Journal of Tropical Medicine and Hygiene as Tropical Medicine and International Health, Quarterly Journal of Medicine as QJM: An International Journal of Medicine, and Comptes Rendus Hebdomadaires des Seances de l Academie des Sciences Serie D as Comptes Rendus Biologies.

The most productive author was Grovel PJ who had 7 articles (Table 3). Authors came from 26 countries. Authors from the United States of America (USA) contributed to the highest number of articles with 21 (34%) articles, followed by France, 20 (32%), and Spain, 8 (13%) (Table 4). Of the total articles, 17 (27.4%) were from multinational collaboration.

## 4. Discussion

Our results provide a clear picture of the main cited articles in brucellosis research publications history. For example, in the group of genome sequencing, we find at positions 1, 2, 5, 7, and 22 the articles that reported the complete genome sequence for* Brucella melitensis*,* Brucella suis*,* Brucella abortus* strain 9-941,* B. abortus* strain 2308, and* Brucella ovis*, respectively. At position 11, we find the first report of sequencing a vaccine strain (*B. abortus* S19) by Crasta et al. in 2008. The most recent article by Tae et al. in 2011 (position 8) reported on the resequencing of* B. suis*.

In the group of articles on new species identification, we find the following: at position 38, Foster et al. in 2007 studied small, Gram-negative coccobacilli resembling* Brucella* bacterial strains that have been reported from marine mammals since the mid-1990s. The study led to description of two novel species:* Brucella ceti* and* Brucella pinnipedialis*. At position 52, Scholz et al. in 2008 described two strains of* Brucella microti* as novel species. The strains had been originally isolated from clinical specimens of diseased wild common voles* (Microtus arvalis)* during an epizootic in Czech Republic [24]. At position 54, in 2003, Sohn et al. published the first report of community acquired human infections with marine mammal-associated* Brucella* in two patients. Both patients were young men from Peru and the route of infection was not discovered [25]. At position 20, Verger et al. in 1985 challenged the separation of* Brucella* into different species and proposed that several biovars should be placed under a single species only:* B. melitensis* [26, 27].

In the group of articles on molecular diagnostic tests, we find the following: Bricker et al. (position 18, in 1994) described a PCR assay that can identify and differentiate most* Brucella* species and biovars found in the United States. Prior to this assay, PCR assays did not discriminate among species. Baily et al. (position 25, in 1992) developed the first PCR assay around the* Brucella* cell surface protein (bcsp31). This target became one of the most popular targets used in molecular assays [28]. Romero et al. (position 61, in 1992) published a* Brucella* 16S rRNA based PCR assay, and although similar assay was previously described by Herman and De Ridder in 1992 [29], the assay by Romero et al. was taken up more widely [28].

In the group of articles on vaccination, we find the following: at position 13, Schurig et al. in 1991 produced a live attenuated RB51 strain for vaccination. “R” stands for “rough,” “B” for* Brucella*, and “51” for an internal laboratory nomenclature used at the time it was derived. The vaccine has become one of the most commonly used vaccines [30]. At position 21, Renoux et al. in 1971 showed that levamisole treatment* of B. abortus*-vaccinated mice resulted in improved protection from virulent* Brucella* organisms. This finding triggered a flow of papers dealing with experimental and clinical effects of levamisole [31]. Schurig et al., at position 27, and Ko et al., at position 46, present a review on* Brucella* vaccines.

In the group of articles on pathogenicity, we find the following: O'Callaghan et al. in 1999, at position 15, were the first to identify a new member of type IV secretion system family encoded by virB operon in* B. suis* during a screen for virulence factors. They also showed that the system is essential for the intracellular growth during infection [32]. The system is one of few classical virulence factors identified to date [33]. The type IV secretion system is a pumping system that selectively transports proteins or other macromolecules through membranes [34]. After* Brucella* is taken up by vesicles in macrophage, acidification is thought to induce VirB expression. The VirB system interacts with components of the endoplasmic reticulum, neutralising the pH and allowing the* Brucella* to undergo regulated cell division [34]. Other classics that further explored this system include Celli et al. in 2003 (position 16), Comerci et al. in 2001 (position 31), Boschiroli et al. in 2002 (position 34), Sieira et al. in 2000 (position 40), Kohler et al. in 2002 (position 47), and Hong et al. in 2000 (position 50). In addition, Sola-Landa A et al. in 1998 (position 45) identified the BvrR/BvrS system for the first time in* B. abortus* (Bvr:* Brucella* virulence related; R: regulatory; S: sensory). At present, the BvrR/BvrS system is one of the best characterized two component systems. Two component systems allow the bacteria to sense their environment and subsequently modulate the expression of genes [35].

Three large case series appear in the list of classics: Colmenero et al. (position 10), Buzgan et al. (position 36), and Lulu et al. (position 39). The report by Buzgan et al. in 2010 described the clinical manifestations of 1028 cases of brucellosis and was considered to be the largest case series until that time [36]. Other case series in the list were on vertebral osteomyelitis (positions 26 and 33) and neurobrucellosis (positions 35 and 43).

The oldest citation classic article was published in 1950 and was at position 29. It was by Harris who described the side effects associated with the use of aureomycin and chloramphenicol in treatment of brucellosis. Prior to the development of these treatments, chemotherapy of brucellosis yielded unsatisfactory results [37].

The list of classics did not include any article on outbreaks. We suggest the following explanations: (a) papers on* Brucella* outbreaks receive lower citations compared to articles in basic science: in both databases (Scopus and WOS) the highest cited article on brucellosis outbreaks was “Canine Brucellosis: Outbreaks and Compliance, Theriogenology, 2006” (78 citations in Scopus and 72 citations in WOS); (b) outbreaks in* Brucella* have been recognized at very early time; therefore, their findings might have become well known: we found reports of outbreaks as early as 1939 (Water-Borne Outbreak of* Brucella melitensis* Infection. Am J Public Health Nations Health, 1939); (c) identifying* Brucella* outbreaks could be difficult:* Brucella* is difficult to detect and identify [38]; and (d)* Brucella* species are genetically homogeneous, and thus, the typing of* Brucella* species for epidemiological purposes by conventional molecular typing methods has remained elusive [39].

We also observed the lack of papers on brucellosis in animal health and for this we suggest two explanations: (a) journals in the categories of agriculture and food sciences receive fewer citations than those in basic and clinical sciences as evidenced by the impact factor in these categories. For example, in the WOS, in the categories of “agriculture, dairy, and animal sciences” and “food science and technology,” the highest impact factor for a journal was 4.7 and 7.3, respectively. While in the category of “medicine, general and internal” and “microbiology,” the highest impact factor for a journal was 72 and 23.6, respectively. (b) The possible low productivity of research that is performed on* Brucella* as evidenced by the lower number of articles on* Brucella* in agricultural journals. For example, a combined search for the word “brucell^*∗*^” and the journals “Veterinary Research” and “Journal of Dairy Science” yielded 20 and 25 papers, respectively, while the same search in the journals “Clinical Infectious Diseases” and “Journal of Bacteriology” yielded 52 and 237 papers, respectively. Furthermore, we doubt that our search missed important journals from the agricultural fields because the databases Scopus and WOS include large collection of agricultural journals. Scopus has 2608 journals included under the “agricultural and biological sciences” subject area and WOS has 58 journals included under the category “agriculture, dairy, and animal sciences” and 130 journals under the category “food science and technology.”

Studies on citation classics that used more than one databases are few and have ranked the articles according to the mean of the citation counts in the databases [40–43]. Here, we ranked the articles according to the highest obtained citation count from any of the two databases. We believe that our method is more accurate because relying on the mean for ranking might lower the rank of a given article. This is because the databases differ in reporting the citation count for a particular article. The variation in citation count between databases results from differences in journal coverage and quality [44]. Scopus includes a more expanded spectrum of journals than WOS, and its citation analysis is faster and includes more articles than the citation analysis of WOS [45]. However, Scopus tends to miss older citations which results in omission of studies before 1980 [46, 47]. Here, we identified 12 articles that were listed in the highest 50 articles in one database but were not identified within the highest 50 articles in the other database and vice versa. Articles that were identified by WOS and not by Scopus tended to be older and of basic science type, while articles identified by Scopus and not by WOS were more recent and mostly of observational and review type.

We found that many countries had contributed to the classics including American, European, African, and Mediterranean countries (Table 3). This might reflect the epidemiological distribution of* Brucella*. In addition, the finding that the most recent classic article was in 2011 indicates that brucellosis is a dynamic field of study [21, 48–51].

Our study has several limitations that are similar to other studies in citation classics [21]. These limitations include the presence of inherent problems in the citation process itself, for example, incomplete or inappropriate citations, biased citation [44, 45, 52, 53]; changes in the list of citation classics with time making it a snapshot of the current state of research [54]; absence of articles with languages other than English which is mostly because authors are more likely to cite articles in their own language, and English articles are more likely to be cited overall [20]; and finally, missing of important studies because their findings became well known [55]. The latter point is relevant here because brucellosis was discovered in 1887 and it is possible that some important studies were not indexed in current database but their findings are now considered well known. Despite these limitations, the study provides a picture for the main cited articles in brucellosis research publications since the discovery of* Brucella* 130 years ago.

In conclusion, the citation classics in brucellosis were (a) all in English except one in French, (b) contributed by authors from several countries where brucellosis was or is still endemic, (c) mostly of basic science type, and (d) published in relatively high numbers in recent years indicating a dynamic field of study. In addition, we suggest that performing the search in more than one database would detect additional articles.

## Figures and Tables

**Table 1:** Most cited articles in brucellosis.

Rank	Citation count (Scopus)	Citation count (WOS)	Title	Year	Journal	First author
1	371	964	The genome sequence of the facultative intracellular pathogen *Brucella melitensis*.	2002	Proc Natl Acad Sci USA	DelVecchio VG

2	305	916	The *Brucella suis* genome reveals fundamental similarities between animal and plant pathogens and symbionts.	2002	Proc Natl Acad Sci USA	Paulsen IT

3	726	652	Brucellosis: an overview.	1997	Emerg Infect Dis	Corbel MJ

4	717	665	The new global map of human brucellosis.	2006	Lancet Infect Dis	Pappas G

5	189	695	Completion of the genome sequence of *Brucella abortus* and comparison to the highly similar genomes of *Brucella melitensis* and *Brucella suis*.	2005	J Bacteriol	Halling SM

6	637	521	Brucellosis.	2005	N Engl J Med	Pappas G

7	111	604	Whole-genome analyses of speciation events in pathogenic brucellae.	2005	Infect Immun	Chain PS

8	8	586	Revised genome sequence of *Brucella suis* 1330.	2011	J Bacteriol	Tae H

9	508	417	An overview of human brucellosis.	1995	Clin Infect Dis	Young EJ

10	390	309	Complications associated with *Brucella melitensis* infection: a study of 530 cases.	1997	Medicine	Colmenero JD

11	54	379	Genome sequence of *Brucella abortus* vaccine strain S19 compared to virulent strains yields candidate yields candidate virulence genes.	2008	PLoS One	Crasta OR

12	329	353	Human brucellosis.	1983	Rev Infect Dis	Young EJ

13	314	342	Biological properties of RB51; a stable rough strain of *Brucella abortus*.	1991	Vet Microbiol	Schurig GG

14	337	304	Human brucellosis.	2007	Lancet Infect Dis	Franco MP

15	298	328	A homologue of the *Agrobacterium tumefaciens* VirB and *Bordetella pertussis* Ptl type IV secretion systems is essential for intracellular survival of *Brucella suis*.	1999	Mol Microbiol	O'Callaghan D

16	293	310	Brucella evades macrophage killing via VirB- dependent sustained interactions with the endoplasmic reticulum.	2003	J Exp Med	Celli J

17	285	292	*Brucella abortus* transits through the autophagic pathway and replicates in the endoplasmic reticulum of nonprofessional phagocytes.	1998	Infect Immun	Pizarro-Cerdá J

18	291	276	Differentiation of *Brucella abortus* bv. 1, 2, and 4, *Brucella melitensis*, *Brucella ovis*, and *Brucella suis* bv. 1 by PCR.	1994	J Clin Microbiol	Bricker BJ

19	271	250	From the discovery of the Malta fever's agent to the discovery of a marine mammal reservoir, brucellosis has continuously been a re-emerging zoonosis.	2005	Vet Res	Godfroid J

20	240	266	*Brucella*, a monospecific genus as shown by deoxyribonucleic acid hybridization.	1985	Int J Syst	Verger JM

21	-* *-* *-	266	Immunostimulatory effect of an imidothiazole in immunization of mice against infection by Brucella- abortus.	1971	Comptes rendus hebdomadaires des séances de l'Académie des sciences. Série D	Renoux G

22	56	262	Genome degradation in *Brucella ovis* corresponds with narrowing of its host range and tissue tropism.	2009	PLoS One	Tsolis RM

23	222	217	Brucellosis: a worldwide zoonosis.	2001	Curr Opin Microbiol	Boschiroli ML

24	204	210	Subversion of Toll-like receptor signaling by a unique family of bacterial Toll/interleukin-1 receptor domain- containing proteins.	2008	Nat Med	Cirl C

25	204	202	Detection of *Brucella melitensis* and *Brucella abortus* by DNA amplification.	1992	J Trop Med Hyg	Baily GG

26	204	160	Pyogenic, tuberculous, and brucellar vertebral osteomyelitis: a descriptive and comparative study of 219 cases.	1997	Ann Rheum Dis	Colmenero JD

27	197	202	Brucellosis vaccines: past, present and future.	2002	Vet Microbiol	Schurig GG

28	202	183	Brucellosis: a re-emerging zoonosis.	2010	Vet Microbiol	Seleem MN

29	9	201	Aureomycin and chloramphenicol in brucellosis; with special reference to side effects.	1950	J Am Med Assoc	Harris HJ

30	199	174	Evaluation and selection of tandem repeat loci for a *Brucella* MLVA typing assay.	2006	BMC Microbiol	Le Flèche P

31	192	198	Essential role of the VirB machinery in the maturation of the *Brucella abortus*-containing vacuole.	2001	Cell Microbiol	Comerci DJ

32	197	157	Serologic diagnosis of human brucellosis: analysis of 214 cases by agglutination tests and review of the literature.	1991	Rev Infect Dis	Young EJ

33	196	144	Brucellar spondylitis: review of 35 cases and literature survey.	1999	Clin Infect Dis	Solera J

34	167	195	The *Brucella suis* virB operon is induced intracellularly in macrophages.	2002	Proc Natl Acad	Boschiroli ML

35	195	133	Neurobrucellosis: clinical and therapeutic features.	1992	Clin Infect Dis	Mclean DR

36	194	120	Clinical manifestations and complications in 1028 cases of brucellosis: a retrospective evaluation and review of the literature.	2010	Int J Infect Dis	Buzgan T

37	191	189	Virulent *Brucella abortus* prevents lysosome fusion and is distributed within autophagosome-like compartments.	1998	Infect Immun	Pizarro-Cerdá J

38	186	189	*Brucella ceti* sp. nov. and *Brucella pinnipedialis* sp. nov. for *Brucella* strains with cetaceans and seals as their preferred hosts.	2007	Int J Syst Evol Microbiol	Foster G

39	188	153	Human brucellosis in Kuwait: a prospective study of 400 cases.	1988	Q J Med	Lulu AR

40	167	187	A homologue of an operon required for DNA transfer in *Agrobacterium* is required in *Brucella abortus* for virulence and intracellular multiplication.	2000	J Bacteriol	Sieira R

41	133	187	Antigenic S-type lipopolysaccharide of *Brucella abortus* 1119-3.	1984	Infect Immun	Caroff M

42	186	142	Incidence and control of brucellosis in the Near East region.	2002	Vet Microbiol	Refai M

43	184	170	Clinical categories of neurobrucellosis. A report on 19 cases.	1987	Brain	Shakir RA

44	179	141	Recognition and optimum treatment of brucellosis.	1997	Drugs	Solera J

45	174	178	A two-component regulatory system playing a critical role in plant pathogens and endosymbionts is present in Brucella abortus and controls cell invasion and virulence.	1998	Mol Microbiol	Sola-Landa A

46	171	162	Molecular host-pathogen interaction in brucellosis: current understanding and future approaches to vaccine development for mice and humans.	2003	Clin Microbiol Rev	Ko J

47	163	169	The analysis of the intramacrophagic virulome of **Brucella sui**s deciphers the environment encountered by the pathogen inside the macrophage host cell.	2002	Proc Natl Acad Sci USA	Kohler S

48	169	164	Brucella intracellular life: from invasion to intracellular replication.	2002	Vet Microbiol	Gorvel JP

49	65	168	T-independent responses in B cell-defective CBA/N mice to *Brucella abortus* and to trinitrophenyl (TNP) conjugates of *Brucella abortus*.	1978	Eur J Immunol	Mond JJ

50	159	167	Identification of genes required for chronic persistence of *Brucella abortus* in mice.	2000	Infect Immun	Hong PC

51	143	165	*Brucella abortus* 16S rRNA and lipid A reveal a phylogenetic relationship with members of the alpha-2 subdivision of the class Proteobacteria.	1990	J Bacteriol	Moreno E

52	154	160	*Brucella microti* sp. nov., isolated from the common vole *Microtus arvalis*.	2008	Int J Syst Evol Microbiol	Scholz HC

53	156	159	Early acidification of phagosomes containing *Brucella suis* is essential for intracellular survival in murine macrophages.	1999	Infect Immun	Porte F

54	159	147	Human neurobrucellosis with intracerebral granuloma caused by a marine mammal *Brucella *spp.	2003	Emerg Infect Dis	Sohn AH

55	146	157	Identification of *Brucella* spp. genes involved in intracellular trafficking.	2001	Cell Microbiol	Delrue RM

56	141	156	Temporal development of protective cell-mediated and humoral immunity in BALB/c mice infected with *Brucella abortus*.	1989	J Immunol	Araya LN

57	155	125	Detection of brucellae in blood cultures.	1999	J Clin Microbiol	Yagupsky P

58	141	154	In vitro *Brucella suis* infection prevents the programmed cell death of human monocytic cells.	2000	Infect Immun	Gross A

59	154	138	Brucellosis in Sub-Saharan Africa: epidemiology, control and impact.	2002	Vet Microbiol	McDermott JJ

60	153	130	Single-step PCR for detection of *Brucella* spp. From blood and milk of infected animals.	1995	J Clin Microbiol	Leal-Klevezas DS

61	150	143	Specific detection of *Brucella* DNA by PCR.	1995	J Clin Microbiol	Romero C

62	150	141	Diagnosis of brucellosis by serology.	2002	Vet Microbiol	Nielsen K

**Table 2:** List of journals and their impact factor.

Journal title	Frequency	Impact factor (2015)
Ann Rheum Dis	1	12.384
BMC Microbiol	1	2.581
Brain	1	10.103
Cell Microbiol	2	4.46
Clin Infect Dis/Rev Infect Dis	5	8.736
Clin Microbiol Rev	1	16.187
Comptes rendus hebdomadaires des séances de l'Académie des sciences. Série D/Comptes Rendus Biologies	1	1.64
Curr Opin Microbiol	1	6.234
Drugs	1	4.883
Emerg Infect Dis	2	6.994
Eur J Immunol	1	4.179
Infect Immun	7	3.603
Int J Infect Dis	1	2.229
Int J Syst Evol Microbiol/Int J Syst Bacteriol	3	2.439
J Am Med Assoc	1	37.684
J Bacteriol	4	3.198
J Clin Microbiol	4	3.631
J Exp Med	1	11.24
J Immunol	1	4.985
J Trop Med Hyg/Trop Med Int Health	1	2.519
Lancet Infect Dis	2	21.372
Medicine (Baltimore)	1	2.133
Mol Microbiol	2	3.761
N Engl J Med	1	59.558

**Table 3:** Authors with 3 or more articles.

Author	Number of “classic articles”
Gorvel JP	7
Moreno E	6
Liautard JP	5
Foulongne V	4
O'Callaghan D	4
Pizarro-Cerda J	4
Akdeniz H	3
Boschiroli ML	3
Bourg G	3
Boyle SM	3
Bricker BJ	3
Cloeckaert A	3
Comerci DJ	3
Halling SM	3
Kohler S	3
Letesson JJ	3
Lopez-Goni I	3
Ouahrani-Bettache S	3
Ramuz M	3
Sriranganathan N	3
Ugalde RA	3
Young EJ	3

**Table 4:** Countries of authors for “citation classics” articles.

Country	Number of articles *N* = 62 *n* (%)
USA	21 (33.8%)
France	20 (32.2%)
Spain	8 (13%)
United Kingdom	6 (9.7%)
Costa Rica	4 (6.5%)
Argentina	3 (4.8%)
Australia	3 (4.8%)
Belgium	3 (4.8%)
Germany	3 (4.8%)
Sweden	3 (4.8%)
Greece	2 (3.2%)
Kuwait	2 (3.2%)
South Africa	2 (3.2%)
Austria	1 (1.6%)
Brazil	1 (1.6%)
Canada	1 (1.6%)
Czech Republic	1 (1.6%)
Egypt	1 (1.6%)
Israel	1 (1.6%)
Kenya	1 (1.6%)
Mexico	1 (1.6%)
Netherlands	1 (1.6%)
Peru	1 (1.6%)
Saudi Arabia	1 (1.6%)
Switzerland	1 (1.6%)
Turkey	1 (1.6%)
